# Supplementary material for: Factors Influencing the Implementation of a New Pharmacist Prescribing Service in Community Pharmacies
Source: Pharmacy (Basel). 2023 Nov 6;11(6):173. doi: 10.3390/pharmacy11060173 (PMC10661259; doi:10.3390/pharmacy11060173)
Supplement: Supplementary file 1 [file pharmacy-11-00173-s001.zip › pharmacy-2621561-supplementary.pdf]

Supplementary material 1. Interview guide for the semi-structured interviews with pharmacists

1. INTRODUCTION

Dear Sir, Mme,

I would like to thank you for the time and trust you have placed on me to conduct this interview.

The aim of this interview is to find out the factors that you perceive as facilitating or limiting the implementation of the pharmacist prescribing service into your pharmacy practice. On this basis, the roles that facilitators could play in implementing the service will be assessed. Facilitators are trainers who help overcoming the factors limiting such implementation and help to reinforce the factors facilitating the implementation into practice.

About this interview: It will last approximately 1 hour and will be recorded to ensure a faithful transcription of your comments and to enable us to analyze them. Do you agree? Anonymity will of course be respected in the processing and reporting of the results.

About the method: This is a semi-structured interview consisting of questions focusing on your opinion of the factors influencing the integration of the pharmacist prescribing service into your pharmacy practice. Although the form of the interview is pre-written, feel free to express whatever you think about the subject.

If you agree, I'll start the recording so we can begin with the questions.

2. PHARMACIST AND PHARMACY PRESENTATION

• How would you describe the pharmacy where you work (chain, group, independent)?

• What is your "status" in the pharmacy (pharmacist owner/manager, manager, adjoint, etc.)?

• What percentage do you work on?

• In what year did you graduate as a pharmacist and where did you graduate as a pharmacist?

• On average, how many pharmacists and how many pharmacy assistants work in the pharmacy every day?

• In your opinion, does the number of people working per day in the pharmacy influence the implementation of the pharmacist prescribing service in practice? If so, how?

• What are the costs for the service in your pharmacy?

• How was this price set and by whom?

3. OPINIONS ON THE PHARMACIST PRESCRIBING SERVICE, SOURCES OF INFORMATION AND TRAINING

• In your opinion, what are the advantages and disadvantages of the pharmacist prescribing service (in relation to patients, pharmacists and the healthcare system)?

• What tools, media or documentation do you need to use for the pharmacist prescribing service (algorithm, program, etc.)?

• What support(s) do you use to guide your consultation?

• What media do you use to document your consultation?

• How do you rate the quality of these tools/supports? Do they make it easier to integrate the pharmacist prescribing service into your practice?

• In the pharmacy where you work, have the pharmacists and pharmacy assistants been trained for the service?

If yes :

What is this training?

Do you think this training is sufficient?

• How do the pharmacy staff update their knowledge of the service (e.g. continuing education, documentation)?

• What level of knowledge do pharmacy assistants have about pharmacist prescribing service? To illustrate this question, I'm going to give you an example of a situation:

SITUATION EXAMPLE: A patient arrives at the pharmacy and is greeted by a pharmacy assistant. The patient has a condition for which the service would be suitable. Does the assistant know the list of health problems and how to present the service, so that s/he can transfer the case to you for the consultation, or does he dispense an OTC on his/her own?

• Do you feel that the training you have received and the tools/supports available are sufficient, or would you need more knowledge or resources for the pharmacist prescribing service medicines?

o **If you need more:** what would you need?

• In your pharmacy, how did you and the rest of the team initially react to the implementation of the pharmacist prescribing service into practice?

• What influence do you think the pharmacist prescribing service could have on the Swiss healthcare system?

o **If not public health:** and from a public health point of view (population health), what do you think?

4. DIFFICULTIES, TIME AND COSTS

• Do you find it difficult to implement the pharmacist prescribing service in your pharmacy? Why or why not?

• Do you feel that you have enough time to carry out triage, dispense medicines from the service and document the procedures?

• Do you see pharmacy costs as a factor influencing the implementation of the pharmacist prescribing service? Why or why not? (in terms of investment costs, training, etc.).

• The pharmacist prescribing service drug is not reimbursed by the health insurance scheme and is therefore charged to the patient.

o Do you think this influences its use by your pharmacy team?

o Do you think that the cost to patients influences their willingness to accept the service?

5. HOW THE SERVICE IS PROVIDED IN THE PHARMACY

• In the pharmacy where you work, how is work organized regarding the pharmacist prescribing service? How is it integrated into daily practice?

o **If no answer to question:** is there a protocol/strategy for the process of setting up the service?

• How do the relationships/communication between pharmacists and assistants in your pharmacy influence the use of the pharmacist prescribing service?
• Can you give me an example of a situation in which you have had difficulty using the service?

6. PATIENTS ELIGIBLE FOR THE SERVICE AND PATIENT INFORMATION (PROMOTION)
• In your opinion, who are the patients for whom the service is worthwhile?
• How important do you think this service is for the patients of your pharmacy?
    o How many medicines included in the service do you think you have personally supplied in the last 6 months?
    o Do you consider that you have enough patients for whom you can recommend the service?
• Which medication(s) included in the service do you prescribe the most and why?
• What do you think of the medications included in the service?
    o Do the medications included in the service meet your patients' needs?
    o Would you like other medications to be included in the service? And if so, which ones?
• During research carried out at the Unisanté Pharmacy, it was determined that out of the 109 medications included in the service, only 18 were clinically relevant compared to the OTCs available. What do you think?
• Do you have a marketing strategy in place to inform your patients about the pharmacist prescribing service?
• Has your pharmacy set objectives in relation to the use of the service (objectives in terms of revenue; objectives in terms of number of patients)?
    o **If yes:** What are these objectives and how familiar are pharmacy staff with them?
    o **If yes:** Is there regular feedback on the achievement of these objectives with the whole team?
• In the pharmacy where you work, are there any incentives, such as rewards, for providing the pharmacist prescribing service?
    o **If yes:** What influence do these incentives have on providing the pharmacist prescribing service?

7. RELATIONS WITH OTHER PHARMACIES, GPs AND EXTERNAL INCENTIVES
• Are you working with other pharmacies to implement the service?
    o **If yes:** What influence has this collaboration had on the implementation of the service?
• ***FOR CHAINS/GROUPS:*** Does your chain/group implement interventions that influence the integration of the pharmacist prescribing service into your daily practice? (e.g., training, marketing, patient information, etc.).
    o **If so:** what are these interventions and what influence do they have on the implementation of the service?
    o **If no:** would you find it useful if your chain/group took steps to support the implementation of the service?
• What could your chain/group put in place to help you integrate and use the service?
• Have you informed the GPs around you about the pharmacist prescribing service?

o How do your relationships with GPs influence the provision of the service?

o How do you think GPs feel about the possibility getting medications without prescription in the pharmacy?

• How do you perceive the support of the health authorities and professional associations (at federal and cantonal level) for the implementation of the service?

• Do you feel any external pressure to provide the service?

o **For example:** Does the fact that other pharmacists provide the service influence its use in your pharmacy? If so, how?

8. STAKEHOLDERS INVOLVED IN THE IMPLEMENTATION OF THE PHARMACIST PRESCRIBING PRACTICE

• Is anyone in your pharmacy responsible for this service? (a person responsible for promoting the service or training staff) (project manager, team leader)

o **If yes:** what is this person's function (e.g., managing pharmacist, lead pharmacist, adjoint, etc.) and what roles does he/she play in the provision of the pharmacist prescribing service?

• Are people from outside the pharmacy (such as trainers, facilitators, etc.) involved in implementing the pharmacist prescribing service in your pharmacy?

o **If so:** who are these people and what do they do? How do they influence the provision of the service?

9. THE BENEFITS OF FACILITATORS

• For the pharmacist prescribing service or other pharmaceutical services: have you ever had someone to support you and monitor the implementation of the service?

o **If so:** what kind of help did this person give you? did you find it useful?

• For the pharmacist prescribing service or other pharmaceutical services: would you find useful if someone helped the pharmacy where you work to implement the services?

o **If so:** what kind of help would you need?

10. CONCLUSION

This is the end of our interview. Thank you very much for answering these questions and also for your availability. Is there anything you'd like to add? Is there an important point we haven't covered that you'd like to mention?

Finally, you told me earlier how often you personally provide the pharmacist prescribing service. With regard to the pharmacy where you work, do you know how many medications included in the service have been prescribed in the last 6 months?

If the information is unknown: Would you be willing to find out about this and inform me by e-mail in the next few days?

Supplementary material 2. Questionnaire for pharmacy associations in the French-speaking part of Switzerland

Questionnaire for cantonal pharmacy associations in French-speaking Switzerland

1. Have you set up or do you plan to set up **initiatives** (e.g., training for pharmacists or pharmacy assistants, provision of documents) to help pharmacists implement the pharmacist prescribing service into their practice and/or document the dispensing medications included in the service?

Yes☐ No☐

If yes: Can you specify the nature of these initiatives aimed at implementing, supporting or documenting the dispensing these medications?

If no: from 0 (totally useless) to 10 (really important), to what extent do you think such initiatives would help to implement the pharmacist prescribing service in pharmacy practice??

2. Have you put in place a communication strategy to inform patients about the pharmacist prescribing service and to inform them about its costs?

Yes☐ No☐

If yes: What specific measures have you put in place?

If no: : from 0 (totally useless) to 10 (really important), to what extent do you think that such communication strategy would help to implement the pharmacist prescribing service into pharmacy practice?

3. Have you put in place a communication strategy for general medical practitioners to inform them and to explain to them the pharmacy prescribing service?

Yes☐ No☐

If yes: What have you actually put in place?

If no: from 0 (totally useless) to 10 (really important), to what extent do you think that such communication strategy would help to implement the pharmacist prescribing service into pharmacy practice?

4. At the political level (e.g., cantonal health departments), have you taken any action to support the pharmacist prescribing service?

Yes☐ No☐

If yes: What specific measures have you put in place?

If no: from 0 (totally useless) to 10 (really important), to what extent do you think that such interventions would help to implement the pharmacist prescribing service into pharmacy practice?

5. Are you working with pharmaSuisse or any other cantonal pharmacy associations to support the implementation of the pharmacist prescribing service?

In the event that this study leads to a publication, would you consent that we include your answers, bearing in mind that their anonymity cannot be fully guaranteed due to the size of the group?
Yes☐ No☐
